# Targeting cytokine/chemokine signaling to convert immunologically cold tumors into hot: Emerging strategies in cancer immunotherapy

**DOI:** 10.1016/j.apsb.2026.03.017

**Published:** 2026-03-15

**Authors:** Jung Hee Park, Dae Ui Lee, Jongbin Jeong, Kyung Hee Jung, Soon-Sun Hong

**Affiliations:** a Department of Medicine, College of Medicine, Inha University, Jung-gu, Incheon 22332, South Korea; b Program in Biomedical Science & Engineering, The Graduate School, Inha University, Michuhol-gu, Incheon 22332, South Korea

**Keywords:** Cold tumor, Hot tumor, Tumor microenvironment, Cytokine, Chemokine, Immunotherapy, Immune remodeling, Immune cell activation

## Abstract

The field of cancer immunotherapy has undergone significant advancements in recent years, leading to a paradigm shift in treatment methodologies. However, “cold” tumors, characterized by low immune cell infiltration and an immunosuppressive tumor microenvironment (TME), present considerable therapeutic challenges. In contrast to “hot” tumors, which exhibit vigorous immune activity and responsiveness to immune checkpoint inhibitors, “cold” tumors evade immune surveillance through mechanisms such as impaired antigen expression and restricted T-lymphocyte infiltration. This immune evasion is closely linked to the dysregulation of cytokines and chemokines, which shape the TME and orchestrate immune responses. This review delineates the immune escape mechanisms of cold tumors, with particular emphasis on the role of cytokines/chemokines in modulating the TME. Here we will explore advanced therapeutic strategies that employ engineered chemokines/cytokines (*e*.*g*., IL-2 muteins such as Neo-2/15, IL-15/IL-15R*α* complexes, and CAR-T cells expressing CXCL9/10), nanoparticle-based delivery systems (*e*.*g*., lipid nanoparticles, PLGA nanoparticles, and chitosan-based carriers for targeted cytokine/chemokine delivery), and combination therapies. These strategies aim to remodel the TME to enhance immune infiltration. Emerging therapies designed to transform cold tumors into immunologically active phenotypes through the modulation of cytokines and chemokines are discussed. Finally, the review highlights the ongoing challenges and future directions in using cytokine/chemokine modulation to overcome the limitations of current treatments, emphasizing their transformative potential in addressing the unmet needs of cancer immunotherapy.

## Graphical abstract

Cytokine/chemokine-based strategies, including nanoparticle delivery, gene transfer, combination with immunotherapy, and immune cell activation, collectively reprogram cold tumors and enhance anti-tumor immunity.

**Figure:**
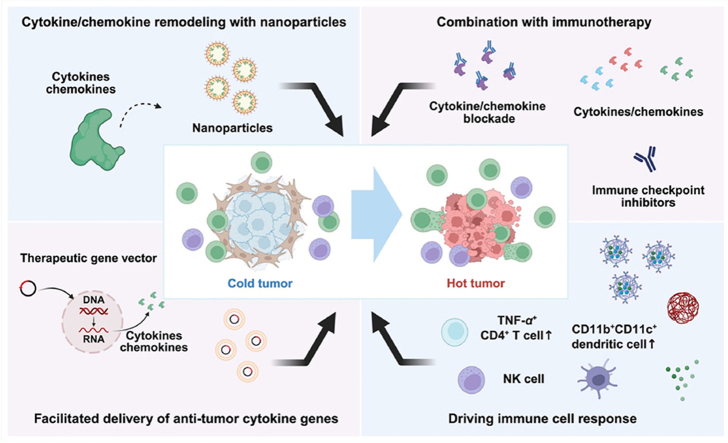

## 1 Introduction

Immune checkpoint inhibitors (ICIs) have become a fundamental component of cancer treatment and are now firmly established in standard oncologic guidelines. Despite the widespread success of ICI therapy, a significant proportion of patients do not respond. This underscores the need for further research and development, particularly in personalized cancer treatment strategies, to improve outcomes for non-responding patient populations^1^. A crucial aspect of advancing cancer immunotherapy is distinguishing between cold and hot tumors based on immune characteristics within the tumor microenvironment^2^. This classification system is defined by three key parameters^3^: (1) tumor-infiltrating lymphocyte (TIL) density, (2) interferon gamma (IFN-*γ*) gene expression signatures, and (3) chemokine profiles^4^^,^^5^. Hot tumors (immune-inflamed phenotype) are characterized by a high density of CD8^+^ cytotoxic T cell lymphocytes (CTLs) and natural killer (NK) cells (typically >100 TILs/mm^2^), robust IFN-*γ*-responsive gene signatures, and elevated expression of pro-inflammatory chemokines such as C–X–C motif chemokine ligand 9 (CXCL9), CXCL10, and CXCL11 that recruit C–X–C motif chemokine receptor 3 (CXCR3)-expressing T cells^6^. These tumors generally exhibit better responses to ICIs. In contrast, cold tumors are defined by low TIL density (<50 TILs/mm^2^) or the absence of T cells, profoundly reduced or absent IFN-*γ* signatures, and diminished chemokine production, particularly low levels of CXCL9, CXCL10, and CXCL11^5^. There are two distinct phenotypes of cold tumors: immune-desert tumors, which lack lymphocytes due to defective antigen presentation, and immune-excluded tumors, which show T cell accumulation at the margins but fail to infiltrate tumor cores due to physical extracellular matrix (ECM) barriers and immunosuppressive signals. These frameworks enable accurate tumor immunophenotyping and rational therapeutic strategy selection. Transforming cold tumors into hot tumors is a pivotal strategy for enhancing cancer therapy efficacy. Cold tumors, despite their immune-excluded phenotype, possess an underlying capacity for immune activation when appropriate interventions overcome their specific barriers. Novel therapeutic approaches targeting different stages of the cancer-immunity cycle, particularly antigen presentation and immune cell recruitment, are being developed to achieve this transformation.

Recent clinical advances over the past decade have identified three complementary strategies essential for cold-to-hot tumor conversion: 1) enhancing tumor antigen presentation, 2) promoting effector T cell recruitment and trafficking, and 3) overcoming physical barriers within the tumor microenvironment. FDA-approved oncolytic virus talimogene laherparepvec (T-VEC) demonstrated efficacy through immunogenic cell death and major histocompatibility complex (MHC) upregulation, with randomized trials showing synergistic benefits when combined with checkpoint inhibitors^7^, ^8^, ^9^. Personalized neoantigen vaccines have advanced from proof-of-concept to phase 2b trials, earning FDA Breakthrough Designation, alongside dendritic cell vaccines and STING agonists showing promise in priming tumor-specific immunity^10^, ^11^, ^12^, ^13^, ^14^. For T cell recruitment and trafficking, engineered chimeric antigen receptor (CAR)-T cells secreting chemokines such as C–C motif chemokine ligand 19 (CCL19) and CXCL9 achieved high response rates, while CXCR4 antagonists combined with checkpoint blockade enhanced infiltration in glioblastoma (GBM) and hepatocellular carcinoma, and bispecific T cell engagers demonstrated efficacy in overcoming immune exclusion^15^, ^16^, ^17^, ^18^.

Overcoming physical tumor microenvironment (TME) barriers has proven equally critical, with vascular normalization strategies combining anti-VEGF therapy (Bevacizumab) with immunotherapy (Atezolizumab) showing superior efficacy in landmark trials, including IMmotion151 in renal cell carcinoma (RCC) and IMbrave150 in hepatocellular carcinoma (HCC)^19^^,^^20^. Transforming growth factor-beta (TGF-*β*) inhibitors enhanced T cell penetration in fibrotic tumors, FAP-targeted therapies disrupted stromal barriers, and radiotherapy combined with immunotherapy induced abscopal effects through TME remodeling^21^, ^22^, ^23^, ^24^, ^25^. These diverse strategies converge on a central principle whereby all rely on modulating cytokine and chemokine signaling to convert cold tumors. Namely, oncolytic viruses induce type I interferons and CXCL9/CXCL10, and therapeutic vaccines stimulate IFN-*γ* and interleukin-2 (IL-2). Engineered T cells deliver immune-stimulatory cytokines and chemokines, and VEGF blockade upregulates endothelial chemokine receptors. Additionally, stromal remodeling restores chemotactic gradients that guide T cell infiltration. As cytokine and chemokine signaling is the unifying biochemical process that drives successful tumor conversion, this review comprehensively focuses on strategies for modulating these pathways and transforming cold tumors into immunologically responsive states.

Cytokines/chemokines are small protein molecules secreted by both immune and non-immune cells, serving as critical messengers for intercellular communication. They play multifaceted roles in the cancer immunity cycle, contributing to cancer antigen presentation, T cell priming and activation, effector T cell infiltration into the tumor site facilitated by vascular normalization and restored chemotactic gradients, and the subsequent induction of cancer cell death^26^. As cytokines and chemokines are the functional drivers of all successful tumor conversion strategies, it is essential to examine how these can be harnessed for therapeutic purposes. While recent reviews have addressed cytokine signaling pathways broadly or examined protein engineering and nanoparticle delivery systems independently, no comprehensive review has integrated these complementary approaches specifically for cold tumor conversion. This review addresses this gap by examining how dysregulation of major cytokine families (IL-2, IL-6, IL-7, IL-9, IL-10, IL-12, IL-15, TNF-*α*, interferons) and chemokine axes (CXCL5/CXCR2, CXCL9-10-11/CXCR3, CCL2/CCR2, CCL5/CCR5) contributes to the immunologically cold phenotype. We comprehensively review three therapeutic modalities: engineered cytokines/chemokines with reduced systemic toxicity, advanced nanoparticle-based delivery platforms that enable tumor-targeted administration, and rational combination strategies synergizing these approaches with checkpoint inhibitors. Special focus is placed on strategies with clinical potential by connecting preclinical findings with emerging clinical evidence. By integrating these elements, this review offers a perspective for developing next-generation immunotherapies capable of transforming cold tumors into immunologically responsive states.

## 2 Mechanism of immune escape in cold tumors

Distinguishing between cold and hot tumors is imperative for effective immunotherapy, as cold tumors employ diverse immune escape mechanisms that hinder anti-tumor responses. Overcoming the mechanisms of immune escape in cold tumors remains central to effective immunotherapy.

### 2.1 Impaired antigen presentation

**Figure 1:**
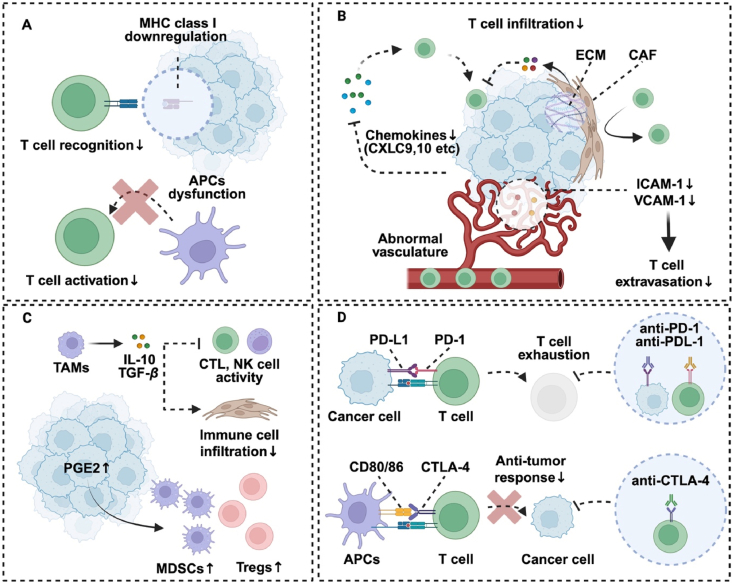
Mechanism of immune escape in cold tumors. (A) Cold tumors impair antigen presentation by downregulating MHC class I, altering antigen processing, and disrupting APC function, promoting immune evasion and limiting T-cell activation. (B) Cold tumors limit immune cell infiltration through reduced chemokine signaling, immunosuppressive CAF activity, a dense ECM, and abnormal vasculature, collectively hindering T-cell access and impairing immunotherapy efficacy. (C) An immunosuppressive cytokine milieu dominated by IL-10 and TGF-*β* suppresses immune activation and impairs antigen presentation; PGE2 further promotes expansion of Tregs and MDSCs, collectively contributing to immune evasion and reduced immunotherapy response. (D) Upregulation of immune checkpoints such as PD-L1 and CTLA-4 in cold tumors leads to T-cell exhaustion and suppressed anti-tumor responses. MHC, major histocompatibility complex; APC, antigen-presenting cell; CAF, cancer-associated fibroblast; ECM, extracellular matrix; TAM, tumor-associated macrophages; CTL, cytotoxic T cell lymphocyte; NK, natural killer; PGE2, prostaglandin E2; MDSC, myeloid-derived suppressor cell; Treg, regulatory T cell.

Antigen presentation is critical for immune recognition and the targeting of cancer cells, which is often impaired in cold tumors, thereby facilitating immune evasion and tumor progression (Fig. 1A). A prevalent mechanism involves the downregulation or loss of MHC class I molecules, which are imperative for the presentation of tumor antigens to CTLs^27^. Furthermore, defects in components of the antigen processing machinery, such as the transporter associated with antigen processing and proteasomal subunits, can further diminish the presentation of tumor-associated antigens^28^. The TME exacerbates these impairments by promoting dysfunctional antigen-presenting cells (APCs), particularly dendritic cells (DCs), which fail to effectively process and present antigens. Consequently, the restoration of antigen presentation has emerged as a promising avenue for immunotherapy. Beyond these MHC class I and antigen processing machinery defects, cold tumors can also exhibit loss or downregulation of MHC class II molecules on both APCs and some cancer cells, critically impairing CD4^+^ T cell activation and limiting adaptive immune response^29^. Tumor-derived immunosuppressive factors, including cytokines and metabolites, can reduce both MHC class I and MHC class II expression on macrophages and DCs, resulting in antigen-presentation failure at multiple levels^30^. Additionally, reduced tumor mutational burden and limited neoantigen load restrict the spectrum of tumor antigens available for T cell recognition, as both the quantity and quality of neoantigens influence immune surveillance, including factors such as clonality and MHC binding strength^31^. These antigen presentation defects produce a self-reinforcing cycle in which impaired neoantigen presentation by both MHC class I and II pathways limits CD8^+^ and CD4^+^ T cell priming, reducing production of the cytokines IFN-*γ* and IL-2 and T cell-derived chemokine CXCL9 and CXCL10 required for immune infiltration into the tumor. Additionally, pro-inflammatory cytokines such as IL-12 and tumor necrosis factor-alpha (TNF-*α*) can promote DC maturation and activation, improving their capacity to process and present tumor antigens^32^^,^^33^. Given this multifaceted dysfunction, therapeutic strategies must target multiple areas simultaneously, such as upregulating both MHC class I and II expression, repairing the antigen processing machinery, and enhancing DCs function. Importantly, these antigen presentations are best combined with modulation of cytokine and chemokine networks to promote T cell infiltration and effective function. Adjusting this approach to the specific defects present in individual tumor types can convert non-responsive tumors (cold tumors) into responsive ones (hot tumors), thereby enhancing the efficacy of immunotherapy.

### 2.2 Lack of immune cell infiltration

A hallmark of cold tumors is the presence of a paucity of CTLs and other effector immune cells (Fig. 1B)^34^. This deficiency significantly impairs the immune system's capacity to recognize and eliminate tumor cells, hindering the efficacy of immunotherapy. A notable contributing factor to this immune exclusion is impaired chemokine signaling, which is critical for the recruitment of immune cells to the tumor site. Tumors exhibiting reduced levels of chemoattractant chemokines, such as CXCL9 and CXCL10, exhibit poor T-cell infiltration, while elevated levels of immunosuppressive factors, such as TGF-*β*, actively repel immune cells^35^^,^^36^. The cancer-associated fibroblasts (CAFs) in the TME also play a pivotal role by secreting immunosuppressive cytokines and generating a dense ECM. In cholangiocarcinoma, CAFs physically impede T cell infiltration, promoting immune evasion^37^. Similarly, stromal desmoplasia in pancreatic cancer and triple-negative breast cancer (TNBC) restricts immune cell access due to ECM rigidity and organization^38^^,^^39^. In addition, the presence of abnormal tumor vasculature contributes to immune exclusion by impairing immune cell trafficking and survival. Dysfunctional endothelial cells in the tumor vasculature frequently manifest a deficiency in adhesion molecules, including ICAM-1 and VCAM-1, which are essential for T-cell extravasation. In certain instances, these aberrant blood vessels can induce apoptosis of CD8^+^ T cells, thereby reducing the ratio of CD8^+^ T cells to regulatory T cells (Tregs) and fostering immune tolerance^40^. The resulting absence of TILs hinders immune surveillance and limits the efficacy of immunotherapies. Addressing the paucity of immune cell infiltration necessitates strategies to enhance immune recruitment, remodel the ECM, and normalize tumor vasculature. Emerging approaches, such as TGF-*β* inhibitors, ECM-targeted therapies, and chemokine-based treatments, have demonstrated potential to overcome immune exclusion and enhance patient responses to immunotherapy. Cytokine and chemokine-based strategies hold particular promise in addressing the lack of immune cell infiltration characteristic of cold tumors. Restoring T cell recruiting chemokines such as CXCL9, CXCL10, and CXCL11 can facilitate CTL migration to the tumor *via* CXCR3 signaling and enhance immunotherapy outcomes^41^^,^^42^. At the same time, modulating the TGF-*β* pathway with inhibitors or neutralizing antibodies can disrupt excessive ECM production by CAFs, thereby improving immune cell accessibility to tumor sites^43^^,^^44^. Pro-inflammatory cytokines, including IL-12 and IL-15, support the expansion and trafficking of effector T and NK cells into tumors. Combination therapies that enhance chemoattractant signals, such as CXCL9 and CXCL10, while simultaneously suppressing immunosuppressive cytokines, have increasingly demonstrated the potential to convert cold tumors into immune-infiltrated hot tumors^45^, ^46^, ^47^. Together, these multi-pronged interventions illustrate how coordinated modulation of chemokines, cytokines, and the tumor stroma can overcome immune exclusion and improve the efficacy of immunotherapies.

### 2.3 TME modulation by immunosuppressive cytokines

The TME in cold tumors is often characterized by the predominance of immunosuppressive cytokines, which hinder immune cell function and facilitate tumor progression (Fig. 1C)^48^. Key cytokines, such as IL-10 and TGF-*β*, are commonly secreted by immunosuppressive cells, including tumor-associated macrophages (TAMs) and Tregs^49^. In cancers such as GBM and bladder cancer, IL-10 and TGF-*β* contribute to an immunosuppressive TME that attenuates CTL activity and reduces T-cell proliferation^50^^,^^51^. TAMs, particularly those polarized to the M2 phenotype, are major producers of IL-10 and TGF-*β*, further suppressing effector immune responses and fostering immune escape. In bladder cancer, elevated levels of prostaglandin E2 amplify this effect by promoting the recruitment and expansion of myeloid-derived suppressor cells (MDSCs) and Tregs^51^. TGF-*β* also impairs DC maturation and antigen presentation, while remodeling the ECM through CAFs, limiting immune cell infiltration^36^. Similarly, IL-10 impedes the production of pro-inflammatory cytokines and diminishes the activation of effector T cells and NK cells. These cytokines collectively disrupt the cancer-immunity cycle, thereby reducing immune cell recruitment, activation, and effector function^52^^,^^53^. Addressing this immunosuppressive cytokine milieu requires a strategic shift from immunosuppressive to pro-inflammatory signaling within the TME. Blocking key immunosuppressive cytokines such as IL-10 and TGF-*β* while simultaneously delivering pro-inflammatory cytokines, including IL-12, IFN-*γ*, and TNF-*α*, can reprogram the TME toward an immune-permissive state^54^. This combination approach is particularly effective because neutralizing IL-10 and TGF-*β* alone removes the brakes on anti-tumor immunity, while concurrent delivery of pro-inflammatory signals actively accelerates T cell activation, expansion, and infiltration. Additionally, targeting the CCL2/C-C motif chemokine receptor 2 (CCR2) chemokine axis represents a complementary strategy that reduces recruitment of immunosuppressive cells, including MDSCs and M2-polarized TAMs, which are major sources of IL-10 and TGF-*β* within the TME^55^^,^^56^. By simultaneously disrupting immunosuppressive cytokine production through MDSCs and TAMs depletion while enhancing pro-inflammatory signals, this multi-pronged approach creates a feedback loop that perpetuates immune activation and overcomes the self-reinforcing immunosuppressive state characteristic of cold tumors.

### 2.4 Upregulation of checkpoint inhibition

Tumors frequently exploit immune checkpoint pathways, such as PD-1/PD-L1 and CTLA-4, to evade immune surveillance and suppress T-cell activity (Fig. 1D). The upregulation of PD-L1 on tumor cells and TAMs in the TME leads to T-cell exhaustion and impaired anti-tumor responses. This mechanism has been extensively documented in various cancers, including non-small cell lung cancer (NSCLC) and melanoma. Elevated PD-L1 levels on tumor cells and immune-infiltrating cells are associated with a poor prognosis and tumor progression in these cases^57^. The interaction between PD-1 and PD-L1 impedes effective T cell-mediated toxicity, enhancing immune evasion. This phenomenon is further exacerbated by exhausted T cells within the TME that express high levels of PD-1, thereby limiting their capacity to mount a robust anti-tumor response^58^. Similarly, CTLA-4, an inhibitory receptor expressed on T cells, is overexpressed in various cancers. In addition, the co-stimulatory molecules CD80 and CD86, expressed on APCs, interact with CTLA-4 on T cells, modulating T-cell activation and contributing to immune suppression within the TME^59^. The upregulation of CTLA-4 impedes T-cell proliferation and contributes to the formation of an immunosuppressive TME by promoting the activity of Tregs and limiting effector T-cell responses^60^. Collectively, the upregulation of immune checkpoint molecules such as PD-L1 and CTLA-4 poses a significant obstacle to immune activation, particularly in cold tumors where immune cell infiltration is inherently low. ICIs, including anti-PD-1/PD-L1 therapies (*e*.*g*., pembrolizumab, nivolumab) and anti-CTLA-4 therapies (*e*.*g*., ipilimumab), have exhibited substantial therapeutic benefits by restoring T-cell activity and enhancing immune-mediated tumor control. However, their efficacy in cold tumors remains limited due to the insufficient presence of immune cells^61^^,^^62^. A comprehensive understanding of the tumor-specific mechanisms that drive checkpoint upregulation is imperative to optimize these therapies, including a strategy to enhance immune infiltration alongside checkpoint blockade. Cytokine and chemokine modulation can substantially enhance the efficacy of checkpoint inhibition therapy by addressing the fundamental limitation of cold tumors, such as insufficient immune cell infiltration and activation. Pro-inflammatory cytokines such as IL-12, IL-15, and IFN-*γ* promote immune cell infiltration and effector T cell expansion, directly providing the population of PD-1-expressing T cells that checkpoint blockade requires for the activation^63^. Type I interferons augment this response through dual mechanisms. They enhance anti-tumor immunity *via* the JAK/STAT pathway while simultaneously upregulating PD-L1 expression on tumor cells and immune cells, thereby sensitizing the tumor microenvironment to checkpoint blockade^64^^,^^65^. Critically, chemokines such as CXCL9 and CXCL10 establish a self-amplifying feedback loop by recruiting CXCR3 T cells to tumors, stimulating IFN-*γ* production from these infiltrating T cells, and further enhancing CXCL9/10 expression through IFN-*γ* signaling^66^. As a consequence of this feedback loop, infiltrating T cells upregulate PD-1 expression, which appears paradoxically favorable because it positions these activated effector T cells for optimal engagement with checkpoint blockade^66^^,^^67^. By combining chemokine-mediated immune recruitment and pro-inflammatory cytokine-driven T cell activation with checkpoint inhibitors that counteract the resulting PD-L1/PD-1 upregulation. This approach overcomes the dual barriers of immune exclusion and immune suppression that characterize cold tumors and limit the efficacy of conventional ICI monotherapy^68^^,^^69^.

### 2.5 Tumor-specific escape mechanisms: intrinsic vs. extrinsic

Tumors employ diverse mechanisms to evade immune detection and destruction, adapting to their unique biology and microenvironment. These mechanisms can be broadly classified into intrinsic mechanisms, which stem from tumor cell–autonomous properties and alterations, and extrinsic mechanisms, which arise from the tumor microenvironment and stromal interactions. Intrinsic mechanisms involve direct tumor cell properties that reduce immune recognition^70^. A prevalent strategy is antigenic variation observed in melanoma, in which the downregulation of melanocyte differentiation antigens reduces tumor visibility to CTLs, impeding effective immune responses^49^. Similarly, defects in the antigen presentation machinery, such as *β*2-microglobulin mutations leading to MHC class I loss in colorectal cancer (CRC), further reduce immune recognition and promote immune escape. Tumors such as GBM multiforme secrete immunosuppressive factors, including TGF-*β*, which inhibit T-cell proliferation and function, further weakening anti-tumor immunity^71^. These tumor-intrinsic alterations directly reduce immunogenicity and suppress local immune responses through tumor cell–derived signals^72^. In contrast, extrinsic mechanisms involve tumors modulating the TME to suppress immune activity beyond direct tumor cell properties. For instance, in HCC, NK cells, which are critical for tumor surveillance, demonstrate diminished cytotoxicity through the repression of activating receptors and the upregulation of inhibitory signals^73^. In gastric cancer, CAFs produce a dense ECM that physically restricts T-cell infiltration and prevents immune cell access^74^. Cholangiocarcinoma employs an alternative strategy, recruiting regulatory immune cells such as Tregs, TAMs, and tumor-associated neutrophils through cytokine release to establish an immunosuppressive barrier around the tumor^75^. These stromal and cellular TME-mediated escape mechanisms operate independently of tumor intrinsic properties, representing a distinct layer of immune evasion^76^^,^^77^. These escape mechanisms highlight the complexity of immune evasion and underscore the need for tailored immunotherapies. In this review, the focus will be on cytokine- and chemokine-based strategies to overcome immune evasion and reprogram the immune landscape of cold tumors. The immune evasion strategies employed by cold tumors operate through multiple connected pathways rather than as single mechanisms. Antigenic variation, defects in antigen processing, immunosuppressive cytokine production, stromal barrier formation, and regulatory immune cell accumulation function cooperatively to establish a hostile microenvironment that resists anti-tumor immunity. This integration of intrinsic and extrinsic escape mechanisms creates redundant defensive networks that require correspondingly comprehensive therapeutic approaches. Importantly, effective treatment extends beyond targeting individual evasion mechanisms to exploit the dependencies and cross-talk between them. For example, the elimination of physical obstacles to immune infiltration and the removal of a principal source of immunosuppressive signaling molecules is achieved by degrading stromal barriers produced by CAFs^78^. Similarly, interrupting Treg recruitment through chemokine axis blockade eliminates immunosuppressive cell populations while simultaneously expanding available space for effector T cell accumulation and function^79^^,^^80^. Therefore, understanding the relative contribution of intrinsic tumor properties versus extrinsic microenvironmental factors is critical for rational therapeutic design. The immune landscape of each tumor should be characterized in detail to provide optimal treatment strategies. It is crucial to identify which evasion mechanisms are predominant, understand the microenvironment composition and tissue architecture, and measure specific cytokine and chemokine signals that maintain immune suppression. With this understanding, modulation of cytokines and chemokines emerges as a key therapeutic principle that can simultaneously overcome immune resistance by addressing both intrinsic and extrinsic evasion mechanisms and converting tumor barriers into opportunities for immune activation in cold tumors.

## 3 Cytokines/chemokines in immunotherapy

As described in the previous section, cold tumors evade immune surveillance through multiple mechanisms, including defective antigen presentation, impaired immune cell infiltration, and the establishment of an immunosuppressive tumor microenvironment. Given that cytokines and chemokines are central regulators of immune cell activation, trafficking, and communication, their modulation provides an attractive strategy to counteract these escape pathways. In this section, we summarize how individual cytokines and chemokines, either alone or in engineered and delivery-optimized forms, can reshape the tumor microenvironment, enhance immune infiltration, and synergize with immune checkpoint blockade to overcome immune resistance in cold tumors.

### 3.1 IL-2 family

The IL-2 family of cytokines includes IL-2, IL-7, IL-9, IL-15, and IL-21, which are involved in regulating immune responses and hold considerable promise in cancer immunotherapy. These cytokines have the common feature of sharing the gamma chain receptor subunit, which is integral to their receptor complexes and signaling pathways. By engaging the JAK/STAT signaling cascade, this cytokine influences the survival, proliferation, differentiation, and function of immune cell subsets, including T cells and NK cells, both of which are central to anti-tumor immunity^81^^,^^82^. Among those, IL-2 has been extensively utilized in cancer immunotherapy, particularly for metastatic renal cell carcinoma and melanoma^82^. In melanoma, IL-2 promotes the proliferation and activation of effector T cells and NK cells, driving robust anti-tumor responses^83^. However, IL-2 therapy is associated with significant systemic toxicities, such as vascular leak syndrome, which limit its application and necessitate careful patient selection and management^84^. Recent advancements, including the development of modified IL-2 molecules (Neo-2/15) with reduced toxicity and combination strategies involving IL-2 and ICIs, aim to enhance its therapeutic efficacy while mitigating adverse effects^85^^,^^86^ Also, bempegaldesleukin (NKTR-214), a PEGylated IL-2 with biased receptor binding favoring CD122 (IL-2R*β*), has demonstrated strong potential in early clinical studies. It advanced to phase III trials in combination with nivolumab for resected high-risk melanoma, reflecting its clinical promise in immune activation with acceptable safety profiles^87^. However, subsequent phase III trials in advanced melanoma and RCC failed to improve patient outcomes despite acceptable toxicity profiles. These results reveal the complexity of modulating IL-2 pathways effectively and suggest that patient selection and tumor microenvironment characteristics play important roles in determining therapeutic success^88^^,^^89^. Other IL-2 family cytokines, IL-21 drives the proliferation and effector functions of both NK cells and T cells, contributing to anti-tumor immunity through pathways analogous to those of IL-2, yet with a reduced propensity for expanding immunosuppressive Tregs^82^. However, in cold tumors, where immune cell infiltration is minimal, the benefits of IL-2 and related cytokines are less clear. Although IL-2 and its family of cytokines have strong therapeutic potential, their efficacy is contingent upon the immune environment of the tumor, implying that customized strategies tailored to different tumor types are needed.

### 3.2 IL-6

IL-6 is a pro-inflammatory cytokine that plays a multifaceted role in the progression of cancer, the infiltration of immune cells, and therapy response. IL-6 signals *via* the IL-6R and activates the JAK/STAT3 pathway, promoting tumor cell survival and proliferation^90^. Within the TME, IL-6 modulates the recruitment and activity of immune cells, often fostering an immunosuppressive milieu by attracting MDSCs and Tregs, which inhibit effective anti-tumor immune responses^91^. Conversely, the role of IL-6 in recruiting effector immune cells, such as CTLs, is less well established and remains context-dependent^92^. IL-6, which is characterized by high mutational burden and immune infiltration, promotes tumor progression through chronic inflammation and immune evasion, contributing to resistance to ICIs^93^. IL-6 sustains chronic inflammation and activates the IL-6/STAT3 pathway to support tumor survival and immune resistance^94^. Given the pivotal function of IL-6 in the progression of tumors and immune modulation, the targeting of IL-6 pathways has emerged as a promising approach to enhance the efficacy of immunotherapy. The IL-6 blockade improves tumor control and a higher density of CD4^+^/CD8^+^ effector T cells^95^. Preliminary studies have demonstrated the efficacy of combining IL-6 inhibition with ICIs, particularly in tumors with high IL-6 expression and immune resistance^96^. Although IL-6 blockade has been proposed as a strategy to improve the efficacy of ICIs, evidence that it can enhance anti-tumor immunity by recruiting CD8^+^ T cells is not strongly supported in the literature^97^. This underscores the necessity for further research into the context-specific effects of IL-6 and its role in TME formation, with a view to developing tailored therapeutic strategies (Table 1).

**Table 1:** Cytokines/chemokines in immunotherapy.

Cytokine chemokine	Function & effect in TME	Immunotherapy strategy	Ref.
IL-2 family	Stimulate T cell and NK cell survival, proliferation and cytotoxicity *via* JAK/STAT signaling; immune outcome depends on tumor context and cytokine variant.	Neo-2/15 (engineered IL-2 mimetic, early evaluation); NKTR-214 (bempegaldesleukin, phase III-no efficacy in melanoma and renal cancer carcinoma); IL-21 boost effector T cells and NK cells and limits Tregs expansion.	81, 82, 83, 84, 85, 86, 87, 88, 89
IL-6	Promotes tumor growth, angiogenesis; recruits MDSCs and Tregs.	IL-6/IL-6R blockade improve anti-tumor immunity and ICIs efficacy; high IL-6 correlates with poor ICI response and immunosuppressive TME.	90, 91, 92, 93, 94, 95, 96, 97
IL-7	Supports T cell survival and infiltration in immunogenic tumors such as melanoma; promotes anti-tumor immunity.	Recombinant IL-7 for T cell reconstitution and converting cold tumors into hot by promoting immune infiltration.	98, 99, 100, 101, 102, 103
	Supports tumor growth *via* JAK/STAT5 or PI3K/AKT pathway in certain solid tumors and leukemias, especially where IL-7R*α* is aberrantly expressed.		
IL-9	Promotes TRAIL, p21-mediated apoptosis; stimulates Th9, dendritic cells, CD8^+^ T cells in melanoma to enhance immunity.	Context-dependent modulation for harnessing or inhibiting IL-9 in cancer immunotherapy.	104, 105, 106, 107, 108
	Promotes Tregs and mast cells in NSCLC and hematologic malignancies, supporting immune evasion and tumor growth.		
IL-10	Suppresses CTLs, NK cells, and APC function in hepatocellular carcinoma and colorectal cancer; facilitates tumor immune evasion.	IL-10/IL-10R blockade is a promising strategy in immunotherapy.	48, 109, 110, 111, 112, 113
	Stimulates CD8^+^ T cells.	IL-10 promotes tumor rejection	
IL-12	Enhances Th1 responses, CTLs and NK cells activation, and IFN-*γ* production; supports anti-tumor immunity.	Localized or targeted IL-12 delivery (fusion cytokines, gene therapy) to minimize systemic toxicity.	114, 115, 116
IL-15	Enhances survival and cytotoxicity of NK cells and CD8^+^ T cells; promotes immune infiltration.	IL-15-based agents to boost cytotoxicity; synergistic with ICIs; IL-15 superagonist (N-803 approved for bacillus calmette–guérin—unresponsive non-muscle-invasive bladder cancer).	117, 118, 119, 120, 121, 122, 123, 124
TNF-*α*	Promotes CTL and macrophage activation *via* TNRF1 signaling in melanoma, enhancing ICI efficacy.	Localized delivery or TNFR-selective modulation to boost anti-tumor immunity while minimizing systemic toxicity; combination with ICIs.	125, 126, 127, 128, 129, 130, 131, 132, 133, 134
	Induces Tregs expansion and ICIs resistance in suppressive TME.		
IFN-*α*/IFN-*β*	Promotes tumor cell apoptosis, enhances antigen presentation by dendritic cells, and recruits CD8^+^ T cells and NK cells.	IFN-based therapies, ICIs combinations, STING agonists, oncolytic viruses to enhance antitumor immunity.	135, 136, 137, 138, 139, 140, 141, 142, 143
IFN-*γ*	Stimulates antigen presentation and activation of CTLs and NK cells in T cell inflamed tumors and promotes tumor apoptosis *via* JAK1–STAT1 pathway.	PD-L1 or IDO1 blockade to boost anti-tumor immunity and overcome resistance; IFN-*γ* and nivolumab combination (phase I, metastatic solid tumors)	144, 145, 146, 147, 148, 149, 150
	Drives tumor progression and immune evasion *via* ICAM1–PI3K and PD-L1/IDO1.		
CXCL1/2/5–CXCR2 axis	CXCL1 and CXCL2 act as potent chemoattractants for MDSCs and neutrophils, promoting angiogenesis, immune suppression, and tumor progression associated with poor ICI response in glioblastoma, NSCLC, breast cancer, and pancreatic ductal adenocarcinoma.	CXCR2 antagonist to suppress tumor growth and improve chemo/immunotherapy outcomes; high CXCL5 associates with poor response to ICIs.	151, 152, 153, 154, 155, 156, 157, 158, 159
CXCL9/10/11–CXCR3 axis	CXCL9 and CXCL10 promote CTL/NK infiltration and immune activation in melanoma, triple-negative breast cancer, and NSCLC; CXCL11 preferentially acts *via* CXCR3-B/alt isoforms to recruit Tregs and suppresses anti-tumor immunity in NSCLC and melanoma.	Enhances CXCL9/10 signaling to boost ICI response; modulates CXCL11–CXCR3-B signaling to prevent immunosuppression; high CXCL9/10 expression predicts improved response to ICIs.	5,42,160, 161, 162, 163, 164, 165, 166, 167, 168
CCL2– CCR2 axis	Recruits tumor-associated macrophages, MDSCs, and Tregs; promotes immune suppression, tumor growth, and metastasis.	CCR2 blockade or dual inhibition with IL-6 to restore anti-tumor immunity and enhance NK cell activity; high CCL2 associates with poor prognosis and limited ICIs efficacy.	169, 170, 171, 172, 173
CCL5– CCR5 axis	Recruits Tregs, MDSCs, and tumor-associated macrophages; promotes immunosuppression, tumor migration, and metastasis.	CCR5 antagonists (Maraviroc, Leronlimab), CCR2/CCR5 dual antagonists (Cenicriviroc); early-phase clinical trials with ICIs; high CCL5-CCR5 signaling correlates with immune evasion and ICIs resistance.	174, 175, 176, 177, 178, 179, 180, 181, 182

TME, tumor microenvironment; NK, natural killer; Treg, regulatory T cell; MDSC, myeloid-derived suppressor cell; ICI, immune checkpoint inhibitor; NSCLC, non-small cell lung cancer; CTL, cytotoxic T lymphocyte; APC, antigen-presenting cell.

### 3.3 IL-7

IL-7, acting through its receptor IL-7R*α*, is essential for T cell development, survival, and homeostasis, thereby serving as a key cytokine in maintaining immune responses in cancer^98^. IL-7R*α* is expressed on various immune cells, including T cells and dendritic cells, and the IL-7/IL-7R*α* axis has emerged as an important target in cancer immunotherapy. In immunogenic “hot” tumors with high immune infiltration, such as melanoma, IL-7 enhances T cell proliferation and survival, contributing to a more robust anti-tumor response^99^. Conversely, in immunologically “cold” tumors such as pancreatic cancer, where T cell infiltration is restricted, the impact of IL-7 is diminished. Emerging therapeutic interventions that increase IL-7 levels or modulate IL-7R*α* expression are being investigated as strategies to transform cold tumors into hot ones by enhancing T cell activity and infiltration within the TME^100^. Clinical studies have demonstrated the potential of rhIL-7, a recombinant human IL-7, to enhance peripheral T cell numbers and their tumor infiltration, thereby potentiating anti-tumor activity^101^. The dual role of IL-7 is determined by the expression of cell-type-specific receptors. While IL-7 promotes anti-tumor immunity through IL-7R*α* signaling on T cells and DCs, tumor cells in certain malignancies (*e*.*g*., acute lymphoblastic leukemia and some solid tumors) can abnormally express IL-7R and engage IL-7 signaling to activate the JAK/STAT5 or PI3K/AKT pathways, thereby promoting their own survival and proliferation^98^^,^^102^^,^^103^. This context-dependency underscores the importance of evaluating tumor-intrinsic IL-7R expression and carefully balancing IL-7-based immunotherapeutic strategies according to tumor type and the composition of the tumor microenvironment^98^.

### 3.4 IL-9

IL-9 is a pleiotropic cytokine produced by multiple immune cell subsets, including Th9 CD4^+^ T cells, Tc9 CD8^+^ T cells, Tregs, and innate lymphoid cells. IL-9 exhibits profound context-dependency in cancer, with effects determined by three critical dimensions: the immunological context (acute versus chronic signaling), the cellular source of IL-9 production (effector *versus* regulatory populations), and the specific tumor type and microenvironment composition^104^^,^^105^. In tumors with robust immune infiltration, acute IL-9 production by Th9 CD4^+^ and Tc9 CD8^+^ cells promote potent anti-tumor immunity through multiple mechanisms. In melanoma, IL-9 secreted by Th9 cells enhances anti-tumor immune responses by upregulating apoptosis-inducing molecules, including TRAIL and p21, which directly trigger tumor cell apoptosis^105^. Th9-derived IL-9 simultaneously recruits and activates DCs and CD8^+^ T cells within the tumor microenvironment, amplifying anti-tumor immune responses and facilitating robust immune surveillance^105^^,^^106^. This coordinated IL-9-mediated activation of both innate and adaptive immunity demonstrates its powerful anti-tumorigenic potential in immunologically permissive contexts. In contrast, chronic IL-9 signaling in immunosuppressive microenvironments drives tumor progression through fundamentally different mechanisms. In NSCLC, which frequently exhibits a cold tumor microenvironment with limited immune infiltration, IL-9 contributes to tumor progression by promoting the survival and expansion of immunosuppressive cell populations, including Tregs and mast cells. Elevated IL-9 levels in certain hematologic malignancies (*e*.*g*., T-cell lymphomas) also correlate with increased tumor cell proliferation and survival, highlighting IL-9's pro-tumorigenic potential in contexts where regulatory populations predominate. This chronic IL-9 signaling fosters immune evasion by expanding immunosuppressive cellular compartments rather than enhancing anti-tumor effector responses^107^^,^^108^. The opposing effects of IL-9 are governed by two interrelated factors: the target cell compartment and the kinetics of cytokine exposure. Acute IL-9 produced by Th9/Tc9 cells augments antitumor immunity by activating DCs maturation and potentiating CD8^+^ T-cell effector function, in part *via* induction of TRAIL and p21; mast cells can further amplify recruitment of effector leukocytes. In contrast, chronic IL-9 signaling within immunosuppressive tumor microenvironments promotes Tregs differentiation and expands mast cells with regulatory phenotypes, reinforcing immune escape. Thus, the cellular and temporal context determines whether IL-9 is immunostimulatory or immunosuppressive. Therapeutically, approaches that leverage acute, effector-enhancing IL-9 while limiting chronic, regulatory IL-9 may improve responses to cancer immunotherapy.

### 3.5 IL-10

IL-10 is a multifaceted cytokine that exerts context-dependent functions in cancer progression and immunotherapy. It inhibits pro-inflammatory cytokine production and antigen presentation, suppressing CTLs and NK cells, thereby promoting an immunosuppressive TME^48^^,^^109^. In HCC and CRC, elevated IL-10 levels enhance Tregs activity, suppressing CD8^+^ T-cell cytotoxicity and fostering immune evasion^109^^,^^110^. In CRC, this immunosuppressive environment contributes to tumor progression, resistance to therapy, and the maintenance of a cold TME^110^. Consequently, the targeting of IL-10 may emerge as a promising immunotherapy strategy. Conversely, under certain conditions, IL-10 can activate CD8^+^ T cells and promote tumor rejection, although this phenomenon is less well-established^111^. The paradoxical effects of IL-10 arise from cell type-specific patterns of receptor expression and downstream signaling. In myeloid-derived antigen-presenting cells and NK cells, IL-10 induces STAT3 activation, which suppresses their effector functions and facilitates tumor immune evasion^112^. In contrast, tumor-resident CD8^+^ T cells that express high levels of IL-10R respond to IL-10 through preferential activation of STAT1 and STAT5, resulting in enhanced proliferation and cytotoxic activity^111^^,^^113^. These divergent signaling pathways provide a mechanistic basis for the dual role of IL-10 in cancer immunity: suppression of innate immune responses coupled with selective enhancement of adaptive anti-tumor immunity. While IL-10 predominantly maintains an immunosuppressive environment, its modulation could potentially enhance immune responses when combined with other therapeutic modalities. However, further studies are needed to clarify the dual role of IL-10 and to identify specific contexts in which its therapeutic modulation might optimize anti-tumor responses.

### 3.6 IL-12

IL-12 is a pro-inflammatory cytokine that serves as a central link between innate and adaptive immunity, positioning it as an essential component of anti-tumor immune responses. It promotes the differentiation of naïve T cells into Th1 cells, leading to the activation of CTLs and NK cells, both of which are critical for tumor eradication^114^. By enhancing the production of IFN-*γ*, IL-12 boosts the cytolytic functions of CTLs and NK cells, facilitating tumor cell lysis and improving immune-mediated tumor clearance^114^. Moreover, it upregulates the expression of MHC molecules on tumor cells, thereby enhancing antigen presentation and immune recognition^115^. The cytokine's capacity to amplify Th1 responses and boost the activity of CD8^+^ T cells and NK cells makes it particularly effective in inflamed, “hot” TME. However, the systemic administration of IL-12 often leads to severe toxicities, further constraining its therapeutic utility^116^. To overcome these limitations, innovative delivery strategies have been developed to localize IL-12 activity within the TME, minimizing adverse effects while maximizing therapeutic efficacy. These include tumor-targeted IL-12 fusion proteins, viral vectors encoding IL-12, and nanoparticle-based delivery systems designed to enhance the efficacy of IL-12 while reducing systemic toxicity^116^. These findings underscore the potential of IL-12-based therapies to reshape the TME and enhance the effectiveness of cancer immunotherapy across diverse tumor types.

### 3.7 IL-15

IL-15 is a cytokine that supports the development and function of NK cells and memory CD8^+^ T cells, thereby enhancing their cytotoxic activity against tumor cells in tumors with high immune infiltration^117^. The IL-15 signals through a receptor complex consisting of IL-15R*α*, IL-2/IL-15R*β*, and the common gamma chain, facilitating a distinctive *trans*-presentation mechanism. In this process, IL-15 bound to IL-15R*α* on antigen-presenting cells is presented to neighboring NK and T cells, inducing their activation and expansion^118^. IL-15 enhances immune cell infiltration and cytotoxic activity within the TME, rendering it particularly effective in immunogenic “hot” tumors such as melanoma. In these tumors, IL-15 enhances the activity of both NK cells and cytotoxic T cells, contributing to the eradication of tumor cells^119^. Clinically, IL-15-based formulations have rapidly advanced, with IL-15 superagonist such as N-803 (ALT-803) and IL-15/IL15R*α* complexes being evaluated across multiple trials^120^. N-803-based therapy has demonstrated clinical benefit in Bacillus Calmette–Guérin unresponsive non–muscle-invasive bladder cancer, leading to subsequent regulatory approval (Anktiva®), ongoing phase II/III evaluations^121^^,^^122^. Clinical trials have demonstrated the potential of IL-15-based therapies, including recombinant IL-15 and IL-15/IL-15R*α* complexes, to enhance anti-tumor immune responses when used alone or in combination with ICIs. However, the efficacy of IL-15 may be constrained in immunologically “cold” tumors, such as prostate cancer, characterized by low TMB and poor immune infiltration^123^. In such cases, the immunosuppressive TME exerts a limiting effect on the activity of IL-15, underscoring the necessity for combination strategies. The present research endeavors are focused on the optimization of IL-15-based treatment protocols to maximize its immunostimulatory capabilities and address the challenges posed by diverse tumors^118^^,^^124^.

### 3.8 TNF-α

TNF-*α* transmits signals through two distinct receptors with opposing immunological consequences: TNF receptor 1 (TNFR1) and TNF receptor 2 (TNFR2). TNFR1 signaling predominantly mediates anti-tumor effects through NF-*κ*B-induced tumor cell apoptosis and immune cell activation, particularly in macrophages and CTLs. In contrast, TNFR2 signaling can promote Tregs expansion and immunosuppression^125^. This receptor-specific dichotomy means TNF-*α* can simultaneously activate anti-tumor and pro-tumor pathways depending on which receptor predominates in a given cellular context. In immunologically hot tumors with robust CTL infiltration, TNF-*α* predominantly signals through TNFR1-expressing immune cells, enhancing tumor cell killing and immune surveillance. In melanoma with high immune infiltration, TNF-*α* enhances the activation of immune cells and contributes to tumor rejection, particularly when combined with immune ICIs^126^. Conversely, TNF-*α* has been shown to promote tumor progression in immunologically cold tumors dominated by myeloid cells and regulatory populations by driving immune checkpoint upregulation (particularly PD-L1) and therapy resistance^127^. This tumor type-specific microenvironmental context determines whether TNF-*α* promotes or inhibits anti-tumor immunity. In cold tumors, TNF-*α* contributes to immune evasion through multiple mechanisms. In CRC, the TNF-*α*/TNFR2 axis promotes tumor growth by maintaining chronic inflammation while simultaneously limiting immune cell infiltration^128^^,^^129^. TNF-*α* expands Tregs and enhances their suppressive functions, further inhibiting the activity of cytotoxic immune cells^130^. Additionally, TNFR1 signaling in certain contexts can foster an immunosuppressive tumor microenvironment through recruitment of MDSCs and promotion of an M2-skewed macrophage phenotype^131^^,^^132^. Also, TNF-*α* has been shown to promote tumor progression by shaping an immunosuppressive TME, particularly through TNFR1 signaling, while also contributing to the resistance to anti-PD-1 drugs in melanoma^133^. In immunologically permissive contexts, TNF-*α* upregulates adhesion molecules and chemokines and facilitates the recruitment of immune cells, including T cells, to the TME, thereby improving immune cell infiltration and enhancing anti-tumor immunity^134^. Collectively, TNF-*α* demonstrates opposing roles depending on the tumor immune context. In hot tumors with robust TNFR1-positive immune infiltration, TNF-*α* exerts anti-tumor effects, whereas in cold tumors dominated by Tregs and MDSCs, TNF-*α* promotes tumor progression. This dichotomy underscores the critical importance of comprehensive tumor immune profiling before implementing TNF-*α*-based therapeutic interventions. Future therapeutic strategies should selectively leverage TNF-*α*′s immune-activating properties in appropriately immunized or naturally hot tumors. This can be achieved through selective TNFR1 agonists or tissue-targeted TNF-*α* delivery approaches, which would circumvent systemic toxicity while maximizing anti-tumor efficacy.

### 3.9 IFN-α and IFN-β

IFN-*α* and IFN-*β* exert their effects by binding to the interferon-*α*/*β* receptor, activating signaling pathways that lead to the expression of interferon-stimulated genes with antiviral and anti-tumor properties^135^. IFN-*α* received FDA approval for the treatment of malignancies, including melanoma and RCC, due to its capacity to impede tumor cell proliferation and enhance the antigen-presenting capabilities of DCs, potentiating the cytotoxic activity of T cells^136^^,^^137^. Both IFN-*α* and IFN-*β* are instrumental in enhancing immune cell infiltration into the TME. They upregulate chemokines and adhesion molecules, facilitating the recruitment of CD8^+^ T cells and NK cells to tumor sites. This immune infiltration correlates with improved anti-tumor responses, particularly in melanoma and GBM, where IFN-*β* enhances MHC class I expression on tumor cells, boosting immune recognition and response^138^^,^^139^. In melanoma, IFN-*α* amplifies immune responses by activating cytotoxic lymphocytes and improving antigen presentation, contributing to better survival rates^135^. IFN-*α*-liberated cytotoxic lymphocyte capacities exhibited an improvement in the anti-PD-1-induced immune response in HCC^140^. Clinically, IFN-*α* has been extensively evaluated in combination regimens to enhance its anti-tumor efficacy. PEGylated IFN-*α*2b combined with pembrolizumab demonstrated enhanced T cell infiltration and improved response rates in advanced melanoma compared to ICI monotherapy in phase Ib/II trials^141^. In HCC, PEGylated IFN-*α* formulations combined with anti-PD-1/PD-L1 antibodies have shown promising results in phase II trials, demonstrating enhanced tumor immune infiltration and clinical responses^142^. However, prolonged exposure to type I IFN also induced immune suppression and resistance mechanisms within the tumor, limiting their therapeutic efficacy^143^. To address these challenges, current research is focusing on combination therapies to harness the benefits of type I interferons while minimizing adverse effects.

### 3.10 INF-γ

IFN-*γ* exerts a multifaceted role in cancer, acting as both a potent anti-tumor cytokine and a possible enabler of immune evasion, depending on the TME. Predominantly produced by activated T cells and NK cells, IFN-*γ* binds to its receptor and activates signaling pathways that upregulate the expression of interferon-stimulated genes, which in turn mediate immune activation and tumor suppression^144^. A notable anti-tumor mechanism of IFN-*γ* involves the upregulation of MHC class I and II molecules on tumor cells. This process enhances antigen presentation, facilitating recognition and destruction by CTLs and NK cells^144^^,^^145^. Furthermore, IFN-*γ* induces the production of chemokines such as CXCL9, CXCL10, and CXCL11, which attract immune effector cells into the TME, increasing immune cell infiltration and enhancing anti-tumor responses^146^. At elevated concentrations, IFN-*γ* activates the JAK1-STAT1-caspase pathway, leading to apoptosis and increased CTL infiltration^147^. Clinically, a phase I dose-escalation study combining IFN-*γ* with nivolumab in metastatic solid tumors showed acceptable safety with preliminary signals of activity, providing early clinical support that IFN-*γ* pathway engagement can be leveraged in combination immunotherapy^148^. Conversely, low IFN-*γ* levels promote tumor progression by inducing cancer stem-like properties through the ICAM1–PI3K–Akt–Notch1 pathway. Despite its anti-tumor potential, IFN-*γ* can also contribute to immune evasion by upregulating immunosuppressive molecules such as PD-L1 and IDO1, which inhibit T-cell activity^149^. The dual role of IFN-*γ* is determined by acute versus chronic signaling dynamics and tumor cell adaptations: acute IFN-*γ* signaling enhances MHC class I/II expression and antigen presentation on immune cells, promoting tumor recognition and CTL-mediated killing. However, chronic IFN-*γ* exposure in the tumor microenvironment drives adaptive immune evasion mechanisms in tumor cells, including upregulation of PD-L1 and IDO1, which suppress T cell function, and activation of ICAM1-PI3K survival pathways. Additionally, prolonged IFN-*γ* signaling can lead to T cell exhaustion and selection of IFN-*γ*-insensitive tumor cell variants. This temporal context (acute immune activation versus chronic exposure) determines whether IFN-*γ* promotes tumor elimination or facilitates immune escape. Rational therapeutic design should prioritize maintaining the acute IFN-*γ* phase while preventing transition to chronic immune evasion through rational combination strategies. Future clinical trials should employ tumor immune profiling (baseline PD-L1, IDO1, JAK mutation status) to predict which patients will benefit from IFN-*γ*-based therapies alone versus those requiring combination PD-L1 inhibition, IDO1 inhibition, or temporal dosing modification^150^.

### 3.11 CXCL1, -2, -5/CXCR2 axis

The CXCR2 signaling network, which includes ligands such as CXCL1, CXCL2, and CXCL5, plays a pivotal role in cancer progression by promoting angiogenesis, immune cell recruitment, and metastasis within the TME^151^. CXCL1 and CXCL2, in particular, act as chemoattractants for MDSCs and neutrophils, facilitating immune evasion and resistance to immunotherapy^152^^,^^153^. Their effects are context dependent, varying with tumor type and the immune landscape of the TME. In GBM, the elevated CXCL1/2 expression enhances myeloid cell recruitment and suppresses CD8^+^ T cell infiltration, thereby facilitating tumor progression^153^. In pancreatic and CRC models, CXCL1/2-driven myeloid infiltration similarly contributes to immune suppression, whereas blockade of CXCR2 or depletion of CXCL1/2-producing myeloid cells restores T cell infiltration and potentiates the efficacy of ICIs^154^^,^^155^. In NSCLC, elevated levels of CXCL5 expression correlate with a poor prognosis and a diminished response to PD-1 inhibitors^156^. CXCR2 antagonists have been demonstrated to enhance the efficacy of chemotherapies such as gefitinib^157^. In breast cancer, the CXCL5/CXCR2 axis promoted metastasis, and the use of CXCR2 inhibitors improved survival outcomes in patients with HER-2-negative metastatic breast cancer^158^. In pancreatic ductal adenocarcinoma, suppressing the CXCL5/CXCR2 axis reduced tumor growth and enhanced chemotherapy sensitivity by attenuating immune suppression and modulating granulocyte recruitment^159^. Overall, targeting this axis has the potential to improve cancer treatment outcomes by enhancing immune responses and reducing tumor progression.

### 3.12 CXCL9, -10, -11/CXCR3 axis

The chemokines CXCL9, CXCL10, and CXCL11, in conjunction with their receptor CXCR3, play a pivotal role in the migration, differentiation, and activation of immune cells, influencing the efficacy of cancer immunotherapies^5^^,^^160^. Induced by IFN-*γ*, these chemokines bind to CXCR3, which is predominantly expressed on activated CTLs and NK cells. This interaction promotes anti-tumor immunity, which correlates with improved patient outcomes in various cancers, including melanoma, breast cancer, and NSCLC^5^^,^^161^. CXCL9 and CXCL10 recruit CTLs and NK cells, thereby enhancing immune surveillance and amplifying the efficacy of ICIs. TAMs have also been reported to secrete CXCL9 and CXCL10, facilitating CD8^+^ T-cell recruitment *via* CXCR3 and contributing to the efficacy of immune-checkpoint blockade therapies^42^^,^^162^. For instance, in TNBC, these chemokines increase CD8^+^ T cell infiltration, improving tumor immune responses and therapeutic outcomes. Similarly, in NSCLC adenocarcinoma, the CXCL9/10–CXCR3 axis promotes an inflamed TME, improving response to anti-PD-1/PD-L1 therapies^5^^,^^160^. In melanoma, this axis is critical for immune-inflamed tumors, where enhanced expression of both cytokines promotes immune cell infiltration and augments the efficacy of ICI therapy^163^. In contrast, the role of CXCL11 is more complex and context-dependent. Although CXCL11 shares CXCR3 as a receptor with CXCL9 and CXCL10, it preferentially binds to CXCR3-B and CXCR3-alt isoforms. These isoforms initiate distinct downstream signaling pathways, such as STAT3 and AKT activation, which promote Tregs recruitment, upregulate PD-L1 expression, and increase the production of immunoregulatory cytokines rather than activate effector T cells^164^. In some tumors, CXCL11-mediated signaling can drive immune suppression and support tumor progression^160^. Additionally, autocrine or paracrine CXCL11 signaling through these receptor variants can enhance tumor cell migration, induce chemoresistance, and facilitate metastasis by engaging ERK1/2 and other survival pathways^165^, ^166^, ^167^. The balance of CXCR3 isoform expression and downstream signaling thus determines whether CXCL11 exerts immunosuppressive or immune-activating effects within the TME^5^. Thus, depending on the local immune composition and receptor expression, this axis can either impede or enhance anti-tumor immunity and tumor progression. In summary, although CXCL9 and CXCL10 promote the recruitment of immune cells to stimulate effective anti-cancer responses, the immunosuppressive effect of CXCL11 requires careful therapeutic modulation. Therapeutic strategies should aim to amplify the paracrine signaling of CXCL9 and CXCL10, for instance, through gene therapy or fusion protein approaches, while preventing or selectively blocking CXCL11, the autocrine or immunosuppressive actions of CXCL11, potentially by targeting specific CXCR3 isoforms or downstream pathways. Additionally, novel engineered variants of CXCL9 and CXCL10 that resist negative regulation (such as DPP-4 cleavage) have shown promise as next-generation drug candidates^168^. Finally, a personalized approach, based on profiling CXCR3 isoforms and chemokine levels in patient tumors, may optimize responses and minimize unwanted immune suppression or metastasis^166^.

### 3.13 CCL2/CCR2 axis

CCL2 binds to its receptor CCR2 and drives the recruitment of immunosuppressive cells, including TAMs, MDSCs, and Tregs, which collectively inhibit anti-tumor immunity and promote tumor growth^169^. Elevated CCL2 levels have been observed to be associated with increased TAMs infiltration and polarization toward the M2 phenotype in cancers such as esophageal squamous cell carcinoma and breast cancer^170^^,^^171^. In esophageal squamous cell carcinoma, TAMs polarization leads to the expression of PD-L2, contributing to immune evasion by suppressing cytotoxic CD8^+^ T cell responses^170^. In addition to recruiting immunosuppressive cells, the CCL2/CCR2 axis directly influences the function of effector T cells within the TME. Tumor-derived CCL2 has been shown to inhibit T cell activation and promote immune suppression^172^. Notably, tumor cells can express CCR2 themselves, allowing CCL2 to drive tumor proliferation, survival, and metastatic potential through autocrine signaling^171^. Targeting the CCL2/CCR2 axis has emerged as a promising therapeutic strategy, with the potential to disrupt the immunosuppressive TME and restore anti-tumor immunity. Concurrently, dual inhibition of CCR2 and IL-6 has demonstrated the capacity to augment NK cell functionality, particularly in HPV-negative HNSCC^173^.

### 3.14 CCL5/CCR5 axis

CCL5 is a chemokine that is primarily secreted by inflammatory cells, including T cells and monocytes^174^. This axis facilitates the recruitment of Tregs, MDSCs, and TAMs, creating an immunosuppressive TME that promotes tumor progression and evasion of immune surveillance^175^, ^176^, ^177^. In metastatic CRC, increased CCL5/CCR5 signaling correlates with higher infiltration of Tregs and MDSCs, which can lead to resistance to immune checkpoint blockade therapies^178^. In addition, a combination therapy of a CCL5 inhibitor and ICIs has been shown to reprogram the immunosuppressive TME and enhance anti-tumor efficacy in CRC^179^. Similarly, inhibiting the CCL5/CCR5 axis in TNBC reduces immature myeloid cells, underscoring its potential as a therapeutic target^180^. Preclinical studies have demonstrated that CCR5 antagonists, such as Maraviroc, potentiate the effects of ICIs and chemotherapeutic agents by reprogramming the TME to support anti-tumor immunity^181^. CCR5 antagonists, including Maraviroc and Leronlimab, as well as dual CCR2/CCR5 inhibitors such as cenicriviroc, are undergoing early-phase clinical evaluation, often in combination with ICIs or chemotherapy, supporting their translational potential^182^. Thus, targeting the CCL5/CCR5 axis could improve cancer treatment outcomes by impeding the infiltration of immunosuppressive cells and enhancing immune effector responses.

## 4 Overcoming the immune escape of cold tumors: Cytokine-based methods

The use of nanomaterials to remodel cytokines and chemokines has emerged as a promising strategy for converting immunologically “cold” tumors, which have minimal immune cell infiltration, into “hot” tumors that are more responsive to immunotherapy. This approach uses nanoparticles to deliver cytokines or cytokine-encoding nucleic acids directly into the TME, thereby enhancing local immune responses while minimizing systemic toxicity. This targeted delivery recruits and activates immune cells, including dendritic cells and cytotoxic T lymphocytes, which are crucial for effective anti-tumor immunity. These nanomaterials function as localized drug reservoirs, enabling the controlled and sustained release of therapeutic agents. This approach ensures high site-specific efficacy while mitigating the systemic side effects often associated with traditional cytokine/chemokine therapies.

### 4.1 Cytokines/chemokines remodeling with nanoparticle

**Figure 2:**
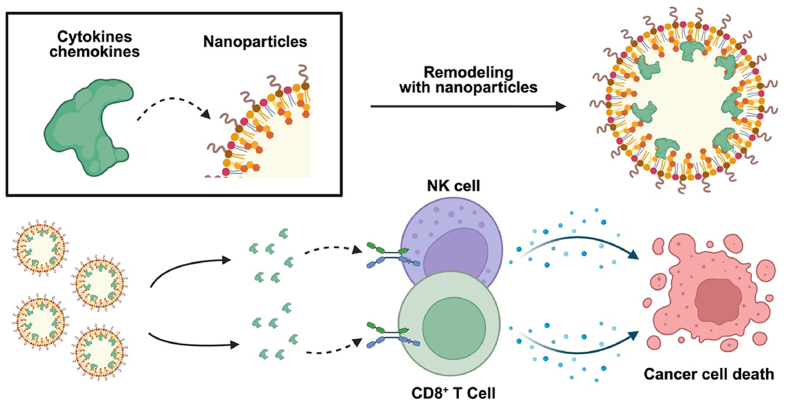
Nanoparticle-mediated delivery of cytokines and chemokines to overcome immune escape in cold tumors. Cold tumors are typically characterized by poor immune cell infiltration and an immunosuppressive tumor microenvironment (TME), which limits the efficacy of immunotherapies. Various nanoparticle-based delivery strategies have been developed to remodel the TME and convert immunologically “cold” tumors into “hot” tumors, enhancing anti-tumor immunity.

A variety of nanoparticles have been used as delivery vehicles for transporting different cytokines and chemokines (Fig. 2). In colon cancer, TNF-*α* encapsulated in PEGylated liposomes enhanced the anticancer effect of radiotherapy by promoting the activation of lymphocytes and NK cells^183^. Naing et al.^184^ have reported that the systemic administration of IL-10 variants, such as PEGylated IL-10 (pegilodecakin), enhances CD8^+^ T-cell-mediated tumor rejection, particularly when combined with ICIs. Moreover, IFN-*γ*-loaded chitosan/*γ*-PGA nanoparticles have been demonstrated to increase cytokine secretion from macrophages, leading to a substantial escalation of IL-6, IL-12, and TNF-*α* levels, while concurrently impeding the invasion and migration of colon cancer cells^185^. Shang et al.^186^ demonstrated that IL-2-loaded nanogels respond to pH in order to target tumor cells and simultaneously release IL-2 and paclitaxel, showing the efficacy of combining chemotherapy and immunotherapy for TNBC. Furthermore, injecting particle-encapsulated IL-2 and IL-12, two cytokines that primarily stimulate a T-cell-mediated immune response, led to a reduction in hepatic and subcutaneous cancer metastases^187^^,^^188^.

In addition to cytokines, chemokines are also being exploited as an innovative approach to regulate the TME and improve the systemic immune response**.** PLGA nanoparticles delivering CCL20 enhanced the regulation of the inhibitory TME and promoted the systemic immune response^189^. Furthermore, targeted editing of CCL5 with CRISPR-Cas9 FCPCV nanoparticles enhanced CD8^+^ T-cell function and cytokine production, including IFN-*γ* and TNF-*α*, and the immune microenvironment, thereby improving breast cancer immunotherapy^190^. Modified magnetic nanoparticles also enhanced CXCR4 and CCR4 expression on NK cells, improving their recognition and cytotoxicity against GBM^191^. Another notable advancement is an implantable alginate/collagen hydrogel encapsulating CCL21-expressing dendritic cells (CCL21-DCs@gel), which has effectively eradicated tumors and prevented tumor recurrence, enhancing the systemic anti-tumor effects of radiotherapy^192^. In parallel with nanoparticle-mediated delivery, cytokine remodeling strategies have been developed to enhance therapeutic selectivity and minimize systemic toxicity^193^. Engineered IL-2 mutein with modified receptor-binding profiles preferentially activate T and NK cells while limiting regulatory T cell expansion, thereby improving anti-tumor immunity with reduced adverse effects^194^. In addition, engineered IL-15 superagonists, such as receptor-concealed IL-15 complexes, have demonstrated durable anti-tumor responses by sustaining CD8^+^ T-cell and NK-cell proliferation and function, as reported in recent preclinical and early clinical studies^195^^,^^196^. Beyond protein engineering, advances in nanoparticle-based technologies have further expanded the cytokine therapeutic strategies. A concealed IL-15/IL-15R complex stabilized within a pH-responsive polymeric nanocapsule enabled tumor-specific activation while minimizing systemic exposure, thereby enhancing safety and efficacy in preclinical cancer models^197^. Similarly, lipid nanoparticle (LNP)-mediated mRNA delivery of cytokines such as IL-15 superagonists has demonstrated potent and durable anti-tumor effects, providing a flexible strategy for spatiotemporal control of cytokine expression^198^. These nanotechnology-driven approaches not only improve pharmacokinetics and tumor selectivity but also synergize with protein engineering to achieve precise modulation of the immune response. These strategies have the potential to fundamentally reshape cancer treatment paradigms by converting cold tumors into hot, responsive sites, offering more effective strategies against diverse cancers.

### 4.2 Facilitated delivery of anti-tumor cytokine/chemokine genes

**Figure 3:**
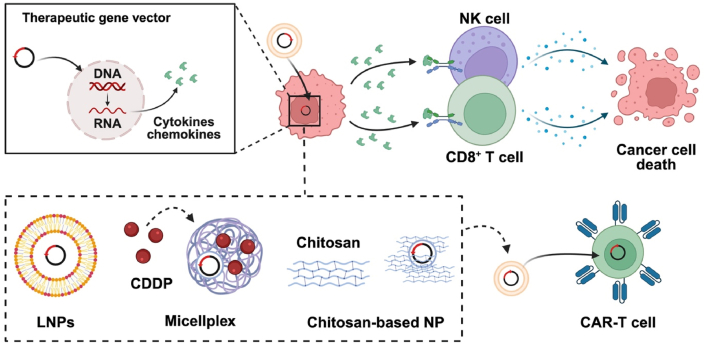
Nanoparticle-based gene delivery strategies using nanoparticles to promote anti-tumor cytokine and chemokine responses. These strategies employ engineered nanoparticles to deliver genes encoding immunostimulatory cytokines and chemokines directly to the tumor site. The resulting local expression of these therapeutic genes amplifies pro-inflammatory signaling within the tumor, thereby enhancing immune-mediated tumor rejection and remodeling the TME. LNP, lipid nanoparticle; CDDP, cisplatin; NP, nanoparticle; CAR-T, chimeric antigen receptor-T cell.

Delivering IL-12 plasmid DNA using bubble liposomes primarily stimulated CD8^+^ T cells and significantly reduced tumor development (Fig. 3)^199^. Furthermore, a highly inflamed TME and primed systemic anti-tumor immunity were also produced by self-replicating RNA-encoded IL-12 encapsulated in LNPs. These effects included high IL-12 expression, IFN-*γ* stimulation, and cancer cell apoptosis^200^. Sun et al.^201^ developed a tumor-targeted micelleplex to co-deliver cisplatin (CDDP) and an IL-12 plasmid (pIL-12), achieving synergistic effects through chemotherapy sensitization and TME modulation in lung cancer. Similarly, administering chitosan-based NPs encapsulating a recombinant pcDNA3.1-dsNKG2D-IL-15 plasmid promoted NK and T cells, resulting in diminished tumor growth and prolonged overall survival of tumor-bearing mice^202^. Novel ionizable lipid materials containing di-amino groups and LNPs encapsulating mRNAs encoding the cytokine IL-27 inhibited tumor growth by inducing the infiltration of immune effector cells into the tumor, including NK and CD8^+^ T cells that produce IFN-*γ* and TNF-*α*^203^. Injectable thermosensitive chitosan hydrogels with CCL5 siRNA reshaped the immunosuppressive TME and improved the efficacy of pancreatic immunotherapies while minimizing systemic toxicity^204^. Additionally, the targeted delivery of a CCL2 trap plasmid (pCCL2) *via* lipid-protamine-DNA nanoparticles enhanced T-cell infiltration and reduced the population of immunosuppressive M2 macrophages, thereby alleviating the immunosuppressive TME^205^. Furthermore, delivering plasmid DNAs encoding CXCL9 and CXCL10 to tumor tissues has been shown to increase T-cell infiltration and improve therapeutic outcomes in preclinical models^206^. Similarly, engineering CAR-T cells to express CXCL9 and CXCL10 has demonstrated superior recruitment of endogenous T cells to tumor sites, resulting in enhanced anti-tumor efficacy^207^^,^^208^.

### 4.3 Combination of immunotherapy with cytokines/chemokines

**Figure 4:**
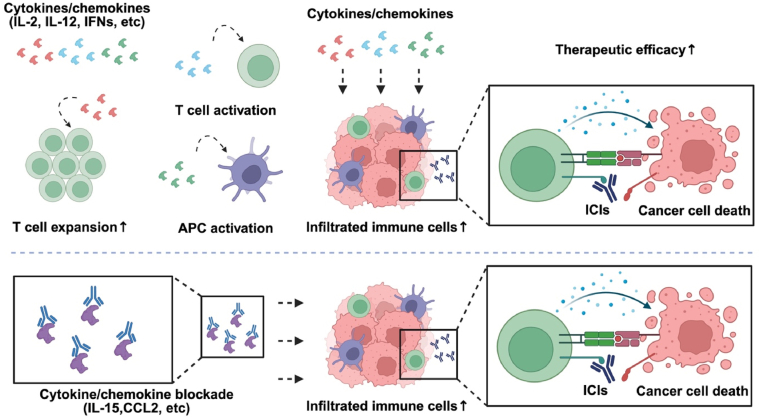
Synergistic effects of combining cytokine/chemokine therapy with immune checkpoint blockade in cancer immunotherapy. Co-administering cytokines/chemokines with immune checkpoint inhibitors (ICIs) elicits a stronger anti-tumor immune response than either approach alone. Cytokine and chemokine therapies recruit and activate immune cells within the tumor microenvironment. Integrating cytokines and chemokines with ICIs has shown robust synergistic effects in enhancing anti-tumor immunity across multiple cancer models.

Combining immunotherapy with cytokines has demonstrated promising synergies in enhancing anti-tumor immune responses (Fig. 4). Interleukins can be combined with ICIs to increase their efficacy. The combination of IL-2 and CTL-4 blockade synergistically enhances therapeutic responses in melanoma by cooperatively activating NK cells and CD8^+^ T cells^209^. In glioma, the combination of IL-12 and CTL-4 blockade primarily targets the CD4^+^ T cell population, resulting in a significant alteration of the immune landscape and a subsequent reduction in the frequency of Foxp3-expressing Treg cells^210^. Also, the combination of TNFR2 blockade with ICIs showed synergistic tumor suppression in various cancer models^211^. Furthermore, a triple combination of anti-CD40/IL-15 and PD-1 blockade enhanced CD8^+^ T cell immune responses in the prostate cancer model^212^. Clinical trials utilizing recombinant IL-15 in combination with spartalizumab further evidence the efficacy by potentiating anti-tumor responses^213^. Combining IL-21 with PD-1 or CTL-4 blockade also promoted CD8^+^ T cell infiltration and proliferation, as well as increased effector memory T cells in mouse tumor models^214^.

Notably, combining PD-1/PD-L1 blockade with IFNs has shown significant potential for enhancing the antitumor immune response^215^. Integrating PD-1/PD-L1 blockers with IFN-*α* amplifies immune cell activation, improves antigen presentation, and increases tumor cell sensitivity, collectively extending patient survival^140^^,^^216^. Furthermore, the combination of PD-1 blockers and recombinant IFN-*α* improved clinical outcomes in unresectable liver cancer by enhancing the efficacy of cytotoxic lymphocytes^217^. Additionally, the combination of IFN-*β* and PD-1 blockade has been observed to elicit an antitumor effect, characterized by increased T cell activation and reduced recruitment of Tregs and TAMs^218^. Nivolumab in combination with IFN-*γ* is also being investigated in clinical trials for advanced solid tumors^148^, underscoring the broad potential applications of cytokines in combination immunotherapy strategies.

In addition to cytokines, chemokines have also been targeted by immuno-oncology drugs. A study combining CCL2 inhibition with immunomodulatory PD-1/PD-L1 targeting produced more favorable results than either monotherapy alone^219^. The combination of CCR2 blockade and anti-PD-1 therapy has demonstrated synergistic tumor suppression and improved survival in tumor-bearing mice^220^^,^^221^. Furthermore, combining anti-CXCL5 and anti-PD-L1 antibodies significantly suppressed the growth of lung cancer, suggesting that CXCL5 facilitates immune escape by upregulating PD-L1 and driving neutrophil chemotaxis through both autocrine and paracrine mechanisms^222^. Recent reports have indicated that promoting the expression of CXCL12 in HCC models induces the recruitment of immunosuppressive cells. The combination of PD-1 and CXCR4 inhibition reduced HCC growth^223^. Additionally, dual CXCR4 and PD-1 targeting maintained the population and activation of TILs in the glioma microenvironment^224^. Similarly, a CXCR4 partial agonist improved immunotherapy by targeting polymorphonuclear myeloid-derived suppressor cells and cancer-driven granulopoiesis^225^. Furthermore, integrating chemokine-based modulation with vessel normalization emerges as a promising strategy to overcome T-cell exclusion in immunologically “cold” tumors. Tumor-associated vasculature remains disorganized, limiting T-cell trafficking despite chemokine gradients^226^. T-cell exclusion is driven by abnormal vasculature expressing low adhesion molecules (ICAM-1, VCAM-1) and elevated immunosuppressive molecules^227^^,^^228^. Vascular normalization strategies combining anti-VEGF therapy (Bevacizumab) with checkpoint immunotherapy have demonstrated superior efficacy in landmark trials^19^^,^^20^. Critically, anti-VEGF therapy restores endothelial ICAM-1/VCAM-1 expression and improves oxygen delivery, creating a permissive window for T-cell extravasation^229^^,^^230^. Concurrently, VEGF blockade upregulates production of CXCR3 ligands (CXCL9-11), which promote robust CD8^+^ T-cell recruitment through newly normalized vessels^230^, ^231^, ^232^, ^233^. This synergy between vessel normalization and chemokine-mediated trafficking is essential for overcoming immune exclusion. Also, inhibition of CCL2 signaling promotes vascular normalization, enhancing nanoparticle delivery and antitumor efficacy^234^. In breast carcinoma models, CCR2 inhibition reprograms the angiogenic and immune microenvironment, reducing tumor growth and invasion^235^. Blockade of the CCL2–CCR2 axis enhances CD8^+^ T-cell infiltration and sensitizes tumors to anti-PD-1 therapy^220^. Similarly, the elevated expression of CXCL10 correlates with intratumoral CD8^+^ T-cell infiltration and induces vascular normalization that improves the efficacy of combined anti-EGFR/PD-1 blockade^236^.

### 4.4 Driving immune cell response for upregulation of anti-tumor cytokines/chemokines

**Figure 5:**
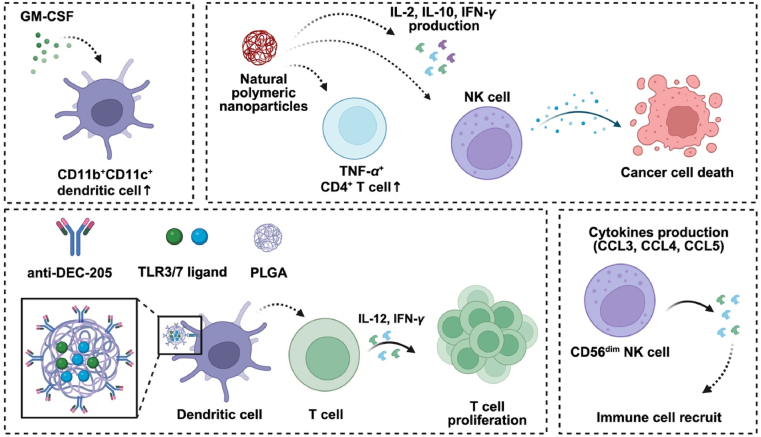
Strategies to drive immune cell responses and upregulate anti-tumor cytokines and chemokines in cold tumors. Biomaterial-based immunotherapy approaches include delivering GM-CSF to expand CD11b^+^CD11c^+^ dendritic cells; natural polymer-based delivery systems, including cyclodextrin to increase TNF-*α*^+^CD4^+^ T cell responses, and chitosan to promote IL-2, IL-10, and IFN-*γ* production in addition to NK cell-mediated cytotoxicity; nanoparticle systems such as PLGA particles co-loaded with anti-DEC-205 and TLR3/7 ligand to enhance dendritic cell-mediated T cell activation; and the role of CD56^dim^ NK cells secreting chemokines, including CCL3, CCL4, and CCL5, which recruit and activate additional immune cells within the TME.

Recent advancements in immunotherapy have focused on developing materials that can actively modulate the immune environment, especially in “cold” tumors with low immunogenicity (Fig. 5). One approach involves using an injectable alginate hydrogel to deliver GM-CSF, which enhances the local recruitment of DCs without inducing their premature activation or maturation. The controlled release of GM-CSF significantly increased CD11b^+^CD11c^+^ DC populations, highlighting the potential of localized delivery systems in shaping immune responses within the tumor site^237^. In parallel, nanoparticle-based platforms offer a promising means to direct and amplify anti-tumor immunity. For instance, Tang et al.^238^ developed PLGA nanoparticles loaded with TLR3/7 ligands and functionalized with DEC-205 antibodies to enhance uptake by DCs. This system stimulated robust IL-12 and IFN-*γ* production by T cells, leading to increased T cell proliferation and a notable 75% survival rate in a mouse lymphoma model. These findings demonstrate that nanoparticles can deliver immune-activating cues directly to antigen-presenting cells, improving overall therapeutic efficacy.

Natural polymers such as cyclodextrin and chitosan have also emerged as effective immune adjuvants. Cyclodextrin has been shown in clinical trials to promote CD4^+^ T cell responses and TNF-*α* production in antigen-stimulated peripheral blood mononuclear cells^239^. Chitosan, widely used as a drug delivery matrix, also exhibits intrinsic immunomodulatory properties. It enhances both humoral and cellular immunity, increases the production of IL-2, IL-10, and IFN-*γ*, and promotes NK cell-mediated tumor cytotoxicity in preclinical studies^240^.

Additionally, the CD56^dim^ subset of NK cells produces significant levels of chemokines such as CCL3, CCL4, and CCL5, which are essential for recruiting and activating other immune cells within the tumor^241^. These chemokines improve immune surveillance and amplify the anti-tumor immune response. Together, strategies ranging from hydrogel- and nanoparticle-mediated cytokine delivery to immune-stimulating polymers and innate immune activation highlight the growing potential of immune cell-targeted interventions. These strategies may improve immunotherapy outcomes by increasing pro-inflammatory cytokine and chemokine production and immune cell trafficking, especially in tumors resistant to immune-based treatments.

### 4.5 Challenges and perspectives (toxicity and delivery-based mitigation)

Although cytokine- and chemokine-based strategies have shown great promise in preclinical models and early clinical trials, there are still significant challenges to overcome before they can be used in clinical practice. The primary limitation is systemic off-target toxicity arising from the short half-lives and widespread tissue distribution of systemically administered cytokines. This broad distribution leads to substantial accumulation in healthy organs such as the liver, kidney, and bone marrow, triggering adverse effects including cytokine release syndrome, vascular leak syndrome (VLS), hepatotoxicity, and multi-organ inflammation that often outweigh therapeutic benefits^242^^,^^243^. High-dose IL-2 exemplifies these challenges, causing severe hypotension, increased vascular permeability, and acute respiratory distress syndrome requiring intensive care management^244^. IL-2-induced VLS specifically results from binding to CD25-expressing endothelial cells, triggering vascular dysfunction and fluid extravasation. Type I interferons (IFN-*α* and IFN-*β*) induce systemic side effects, including flu-like symptoms, fatigue, depression, and hematologic abnormalities that compromise treatment adherence. Even at lower doses, continuous cytokine exposure disrupts homeostatic immune balance, promoting autoimmune manifestations and immunosuppressive feedback mechanisms that paradoxically impede anti-tumor immunity^245^.

Enhanced tumor bioavailability remains elusive despite advanced delivery platforms. The dense extracellular matrix, stromal barriers, and hostile microenvironment of immune-excluded cold tumors severely impede nanoparticle penetration and cytokine diffusion, resulting in inadequate tumor-to-normal tissue concentration gradients^246^, ^247^, ^248^, ^249^. Achieving therapeutic cytokine levels in tumor cores while minimizing peripheral exposure represents an unmet technical challenge. Furthermore, cytokine-based immunotherapy efficacy varies dramatically across tumor types and individuals, reflecting heterogeneity in neoantigen burden, baseline immune infiltration, and tumor microenvironment composition^31^^,^^250^^,^^251^. Cold tumors with pronounced immunosuppression often show limited responses despite cytokine modulation, indicating that uniform approaches are inadequate. Recent advances in protein engineering and delivery technologies offer promising strategies to mitigate these limitations. Engineered IL-2 muteins, such as ALKS 4230, selectively target CD122^+^ immune cells while exhibiting reduced affinity for CD25^+^ endothelial cells, substantially reducing VLS while preserving immunostimulatory activity^252^^,^^253^. PEGylation strategies for interferons extend half-life and reduce dosing frequency, partially mitigating systemic toxicities^254^. For cytokine release syndrome management, next-generation CAR-T designs engineered to produce cytokines locally at tumor sites, biomarker-driven dosing protocols monitoring IL-6 and C-reactive protein levels, and targeted IL-6 receptor antagonism with tocilizumab have improved safety profiles^255^, ^256^, ^257^.

Advanced nanoparticle platforms concentrate cytokine delivery at tumor sites through passive or active targeting mechanisms, reducing systemic exposure. Environmental responsiveness further refines specificity through pH-responsive and protease-activated release systems that activate selectively within the acidic and protease-rich tumor microenvironment. Prodrug linkage strategies exploit tumor-specific enzymatic activity to convert inactive precursors into active cytokines exclusively at tumor sites. Cell-mediated delivery approaches, exemplified by T cells redirected for universal cytokine killing (TRUCKs), concentrate cytokine production directly within tumors, achieving up to 80% reduction in systemic cytokine levels while maintaining robust local immune activation^258^, ^259^, ^260^, ^261^.

These engineered cytokine strategies can partially mitigate systemic toxicity, but continued optimization remains essential. To fully address these challenges, it is necessary to enhance delivery specificity through targeted nanoparticles, develop reversible or inducible cytokine systems that can be rapidly deactivated if toxicity emerges, implement comprehensive immune profiling and biomarker-driven patient stratification, and design tumor-microenvironment-specific combination therapies. Integrating computational modeling, real-time pharmacokinetic monitoring, and personalized medicine approaches is essential to maximize the therapeutic index while minimizing life-threatening adverse events. Ultimately, achieving precise spatial and temporal control of cytokine activity will be key to realizing the full therapeutic potential of next-generation cytokine immunotherapies and expanding their clinical utility across diverse cancer types.

## 5 Conclusions and perspectives

The distinction between “hot” and “cold” tumors has significantly advanced our understanding of tumor immunology and the TME's pivotal role in cancer progression and therapy response. Cold tumors, characterized by poor immune infiltration and an immunosuppressive milieu, pose considerable challenges to conventional treatment modalities. Recent advances have focused on innovative cytokine and chemokine therapies to alter the TME. Engineered cytokines, nanoparticle systems, and mRNA-based delivery platforms facilitate targeted delivery of pro-inflammatory cytokines specifically within tumors while minimizing systemic side effects. Combination therapies integrating cytokines with checkpoint inhibitors have demonstrated synergistic effects that overcome immunosuppressive barriers and transform quiescent tumors into active immune sites.

Future progress in cold tumor conversion will increasingly depend on advanced profiling and computational technologies. Single-cell RNA sequencing and spatial transcriptomics enable the comprehensive dissection of individual tumor immune landscapes by identifying distinct immune cell subpopulations, their functional states, and spatial distributions within specific TME niches, revealing that CD8^+^ T cells occupy tumor cores positioned for killing while stromal immune cells regulate tumor growth^262^, ^263^, ^264^. These technologies, integrated with multi-omics profiling (genomic, transcriptomic, and proteomic), provide comprehensive immune signatures characterizing each tumor's specific deficiencies^265^. Artificial intelligence and machine learning algorithms further accelerate progress by analyzing these complex datasets to predict tumor-specific neoantigens, stratify patients into responders and high-risk groups, and uncover hidden patterns within individual tumors' immune landscapes that guide precision selection of optimal cytokine-based combination therapies^266^^,^^267^. This convergence of high-definition immune profiling, computational intelligence, and personalized medicine enables rational therapeutic design tailored to each tumor's unique immune architecture.

Overcoming cold tumors requires a multifaceted approach that meticulously targets unique immune evasion tactics by integrating emerging technologies. A comprehensive diagnostic pipeline that combines single-cell transcriptomics, spatial transcriptomics, multi-omics profiling, and AI-assisted analysis can generate personalized immune signature profiles, providing direct information on targeted cytokine/chemokine combinations. Ongoing research into detailed immune profiles coupled with technological advances in areas such as cytokine engineering, mRNA therapies, precision delivery, and AI-enabled drug discovery is set to transform therapeutic outcomes. By combining detailed characterization of tumor immune landscapes with innovative analytical approaches and individualized treatment selection, we could achieve sustained responses in cold tumors that have resisted conventional therapy, ultimately transforming the treatment of diverse cancers.

## Author contributions

Jung Hee Park, Dae Ui Lee, and Jongbin Jeong were responsible for writing the original draft. Kyung Hee Jung and Soon-Sun Hong contributed to the conceptualization and to writing-review and editing of the manuscripts.

## AI-assisted technology declaration

The authors used an AI-based language editing tool to improve the clarity and grammar of the manuscript during its preparation. All content was subsequently reviewed and revised by the authors, who take full responsibility for the accuracy and integrity of the work.

## Conflicts of interest

The authors declare no conflicts of interest.
